# Melatonin and Cardioprotection in Humans: A Systematic Review and Meta-Analysis of Randomized Controlled Trials

**DOI:** 10.3389/fcvm.2021.635083

**Published:** 2021-05-12

**Authors:** Alberto Domínguez-Rodríguez, Pedro Abreu-González, Néstor Báez-Ferrer, Russel J. Reiter, Pablo Avanzas, Daniel Hernández-Vaquero

**Affiliations:** ^1^Hospital Universitario de Canarias, Servicio de Cardiología, Tenerife, Spain; ^2^Departamento de Enfermería, Facultad de Ciencias de la Salud, Universidad de La Laguna Tenerife, San Cristóbal de La Laguna, Spain; ^3^Departamento de Fisiología, Facultad de Medicina, Universidad de La Laguna, Tenerife, San Cristóbal de La Laguna, Spain; ^4^Department of Cell Systems and Anatomy UT Health San Antonio, Long School of Medicine, San Antonio, TX, United States; ^5^Área del Corazón, Hospital Universitario Central de Asturias, Oviedo, Spain; ^6^Instituto de Investigación Sanitaria del Principado de Asturias, Oviedo, Spain

**Keywords:** cardioprotection and ischemia-reperfusion injury, melatonin, meta-analysis, human, randomized controlled (clinical) trial

## Abstract

Myocardial ischemia/reperfusion (IR) injury represents a critical problem associated with interventional approaches for coronary reperfusion. Pharmacological cardioprotective interventions are advocated to ameliorate IR injury. Melatonin is an anti-inflammatory and antioxidant agent with a wide range of therapeutic properties that may contribute to its cardioprotective effects. No systematic review or meta-analysis has compared melatonin vs. placebo as a cardioprotective agent in humans. The present study, based on a systematic review and meta-analysis, was carried out to assess melatonin's efficacy as a cardioprotective treatment. We performed a systematic review of the available literature. Randomized controlled trials (RCTs) were identified and information was extracted using predefined data extraction forms. The primary outcomes were (a) left ventricular ejection fraction (LVEF) and (b) blood troponin levels in patients who underwent myocardial revascularization and were randomized to melatonin or placebo. The inverse-variance random-effects method was used to pool the estimates. Heterogeneity and publication bias were assessed. Weighted mean differences or standardized mean differences were calculated. A total of 283 records were screened and seven RCTs met all the inclusion criteria. After the pooled analysis, the results on LVEF were consistent across all studies, and a significant heterogeneity was found in the results on troponin levels. The melatonin-treated patients had on average higher LVEF than the placebo-treated individuals with a weighted mean difference = 3.1% (95% CI 0.6–5.5, *p* = 0.01). Five works compared the levels of troponin after melatonin or placebo treatment. The melatonin-treated patients had lower levels of troponin with a standardized mean difference = −1.76 (95% CI −2.85 to −0.67, *p* = 0.002). The findings of this meta-analysis revealed that melatonin administration in humans as a cardioprotective agent attenuated heart dysfunction with a favorable effect on the LVEF.

## Introduction

Based on epidemiological data, cardiovascular diseases are the number one cause of death globally, causing an estimated 17.9 million deaths each year (1). The most common form of cardiovascular disease, coronary artery disease, has seen a reduction in mortality over the past decades because of reperfusion medical strategies, such as coronary artery bypass grafting (CABG) and percutaneous coronary intervention (PCI). Although timely reperfusion has resulted in a substantial decline in mortality, significant morbidity remains with 22% heart failure at 1 year (2). This is, in part, due to the existence of myocardial ischemia/reperfusion (IR) injury, a phenomenon in which the restoration of coronary blood flows to ischemic myocardium results, paradoxically, in further myocardial injury and cardiomyocyte death (3).

The underlying mechanisms of myocardial IR injury include excessive reactive oxygen species generation, inflammation, and apoptosis. Great effort is needed to identify novel therapeutic strategies regulating myocardial oxidative stress and subsequent inflammation and apoptosis induced by myocardial IR injury (4). Despite numerous cardioprotective agents showing promising results in the experimental setting for preventing myocardial IR injury, their translation into the clinical setting for patient benefit has been challenging, and there is still no effective therapy (5).

Melatonin originally identified in the bovine pineal gland in 1958 (6) has multiple beneficial actions in various cardiac pathologies (7–10). Melatonin is a pleiotropic molecule with several functions that protect the heart against IR injury. It is well-established that melatonin possesses antioxidant and anti-inflammatory activities (7, 11). No systematic review or meta-analysis has been reported comparing melatonin and placebo as a cardioprotective agent in humans. The present study, based on a systematic review and meta-analysis, was carried out to assess melatonin's efficacy as a cardioprotective treatment.

## Methods

### Search Strategy

We conducted and report this systematic review in accordance with the Preferred Reporting Items for Systematic Reviews and Meta-Analyses statement (12) and the Code of Ethics of the World Medical Association (Declaration of Helsinki). The consent of the patients was not required.

In October 2020, MEDLINE through PubMed, Embase, and the Cochrane Central Register of Controlled Trials were used to identify appropriate articles reporting the cardioprotection of melatonin in human myocardial IR injury. The full search strategy was as follows: [Melatonin (Title/Abstract)] AND [myocardial ischemia reperfusion injury (Title/Abstract) OR cardioprotection (Title/Abstract) OR heart disease (Title/Abstract) OR heart (Title/Abstract) OR acute coronary syndrome (Title/Abstract) OR acute myocardial infarction (Title/Abstract)]. Review articles, case report, and editorials were excluded.

### Inclusion and Exclusion Criteria

Studies were eligible for inclusion if they met the following criteria: (a) melatonin compared with placebo; (b) reporting infarct size, expressed as the levels of troponin or left ventricular ejection fraction (LVEF) or the percentage of infarct area evaluated by magnetic resonance imaging; (c) human studies; and (d) randomized controlled trials (RCTs), compared with a control group. We excluded studies investigating the melatonin-mediated cardioprotection *in vitro* or *in vivo* animal studies or publications not written in English.

The primary endpoint was to investigate the potential protective effect of melatonin against myocardial IR injury.

### Data Extraction and Evaluation of the Quality of the Studies

Two members of the team (A.D-R. and P.A-G.) independently examined the title and abstract of the articles, selecting those publications that met the inclusion criteria. Two other members (N.B-F. and R.J.R.) evaluated the previously selected full-text articles and decided their final inclusion on an independent basis. These researchers were blinded to the names of the authors, the institutions, and the names of the journals. Disagreements were resolved through debate among these four team members. The quality of the studies was independently evaluated by two reviewers (P.A. and D.H-V.). To assess the methodological quality of the selected studies, the Cochrane Collaboration's table for assessing risk bias was used (13).

The data extracted from the selected studies were entered in an Excel spreadsheet. Two investigators (A.D-R. and N.B-F.) independently extracted the data sets related to baseline information of the included studies (author, year, and sample size), reperfusion medical strategies (PCI or CABG), the methods for determining the infarct size (levels of troponin or LVEF or magnetic resonance imaging), and detailed therapeutic strategy (route of melatonin administration).

### Statistical Analysis

The inverse-variance random-effects method was used to pool estimates. For LVEF, we used weighted mean differences. Because troponin was reported as I troponin or T troponin, for the meta-analysis of troponins, we used standardized mean differences (14). Heterogeneity was evaluated by Cochran's *Q*-test and by the calculation of *I*^2^. *I*^2^ < 25% indicates low heterogeneity, *I*^2^ 25–75% indicates moderate heterogeneity, and >75% indicates high heterogeneity. For publication bias assessment, we used the Begg test and the Egger test. *p*-values < 0.05 were considered statistically significant. All analyses were performed with the STATA v 16 (StataCorp, TX, USA).

## Results

### Selected Studies

**Figure 1:**
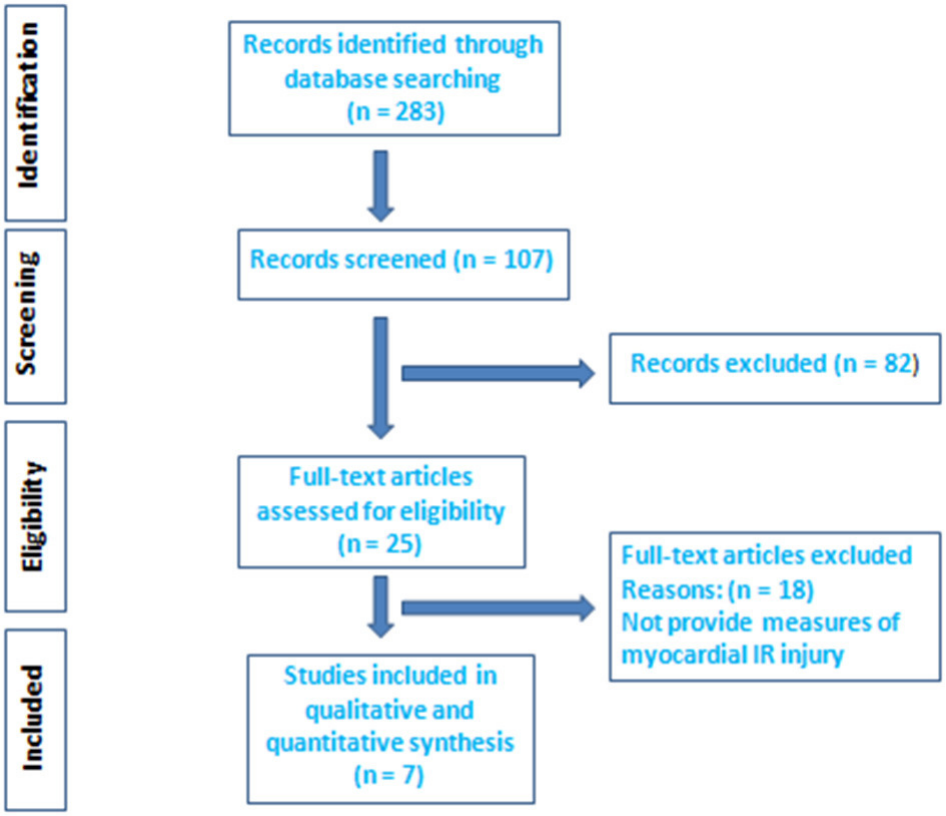
Election of the studies on the basis of the Preferred Reporting Items for Systematic Reviews and meta-analysis flow diagram. IR, ischemia/reperfusion.

Figure 1 shows the study flowchart according to the PRISMA criteria. We evaluated 107 titles and abstracts; 82 were excluded and 25 articles required full-length article evaluation. Eighteen articles were excluded and only seven met all of the inclusion criteria and were included in the qualitative and quantitative pooled analyses (15–21). The total number of patients analyzed was 426. Patients' characteristics of each study are shown in Table 1.

**Table 1:** Overview of the studies included in the meta-analysis.

**References**	**Methods for determining the infarct size**	**Sample size (*n*)**	**Melatonin administration**	**Reperfusion medical strategies**	**Target disease**	**Effect**	**Risk of bias**
Gögenur et al. (15)	Levels of troponin I	50	Melatonin orally and intraoperatively	CABG	Elective abdominal aortic aneurysm repair	CP	High
Ghaeli et al. (16)	Levels of troponin T	40	Melatonin orally	PCI	AMI	No effect	High
Dwaich et al. (17)	LVEF, levels of troponin I	45	Melatonin orally	CABG	CABG	CP	Low
Ekeloef et al. (18)	Levels of troponin T, LVEF, and CRM	48	IV and IC melatonin	PCI	AMI	No effect	Low
Dominguez-Rodriguez et al. (19)	Levels of troponin I, LVEF, and CRM	125	IV and IC melatonin	PCI	AMI	CP in the first tertile (early after symptom onset)	Low
Shafiei et al. (20)	LVEF	88	Melatonin orally	CABG	CABG	CP	Low

*AMI, acute myocardial infarction; CABG, coronary artery bypass grafting; CP, cardioprotection; CRM, cardiovascular magnetic resonance; IC, intracoronary; IV, intravenous; LVEF, left ventricular ejection fraction; PCI; percutaneous coronary intervention*.

### Effect of Melatonin Against Myocardial IR Injury Assessed by Blood Samples and Cardiac Imaging

#### Troponin Levels

**Figure 2:**
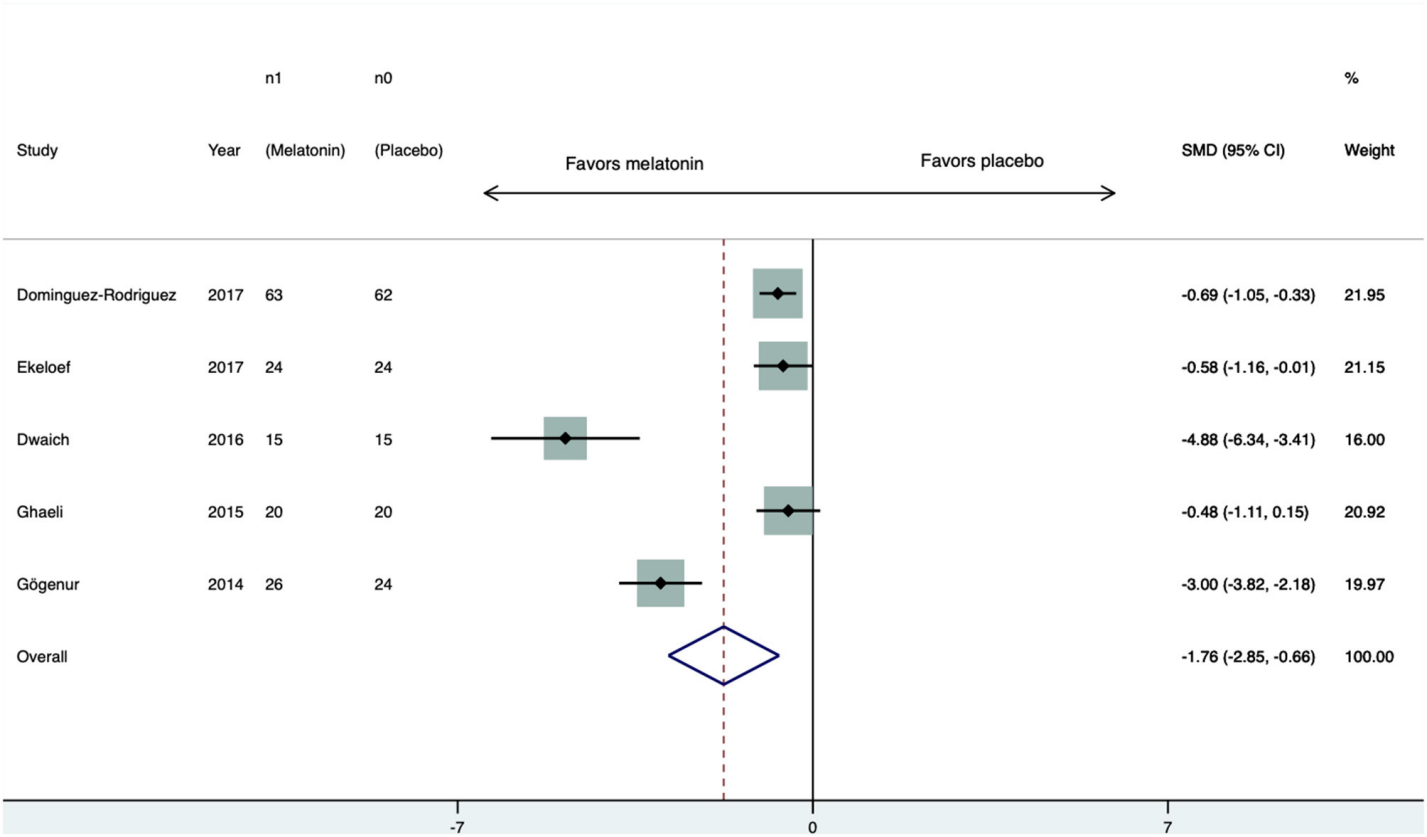
Forest plot of troponin levels. CI, confidence interval; SMD, standardized mean difference.

Five RCTs (15–19) compared troponin levels between melatonin- and placebo-treated individuals. Three (15, 17, 19) of them reported levels of troponin I and two reported levels of troponin T (16, 18). All works reported lower levels of troponin in the melatonin group. The standardized mean difference was −1.76 (95% CI −2.85 to −0.67, *p* = 0.002) (Figure 2). There was substantial heterogeneity among the studies; the *Q* test had a *p*-value < 0.01 and *I*^2^ = 79.8%. There was no sign of publication bias; the Begg test had a *p*-value = 0.8 and the Egger test had a *p*-value = 0.2.

#### LVEF

**Figure 3:**
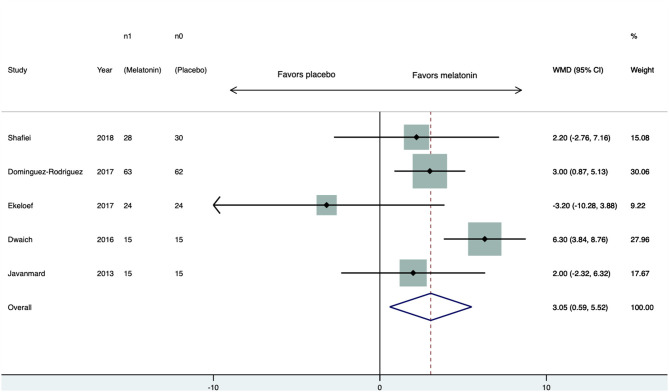
Forest plot of LVEF. CI, confidence interval; WMD, weighted mean difference.

Five studies (17–21) provided values of LVEF. Four studies found higher LVEF in the melatonin-treated patients. After the pooled analysis, the melatonin group had on average 3.1% (95% CI 0.6–5.5, *p* = 0.01) higher LVEF than the placebo group (Figure 3). There was no sign of statistical heterogeneity; the *Q* test had a *p*-value of 0.36 and *I*^2^ = 7.9%. There was no sign of publication bias; the Begg test had *p* = 1 and the Egger test had *p* = 0.24.

#### Magnetic Resonance Imaging

Only two RCTs reported myocardial IR injury (infarct size), assessed by cardiac magnetic resonance imaging (18, 19). Due to the small number of studies, we did not perform a meta-analysis and only the systematic review is presented.

## Discussion

As far as we know, this is the first meta-analysis that provides a comprehensive evaluation of the possible benefits of melatonin in protecting the myocardium against IR damage in humans. To date, only seven RCTs (15–21) investigated melatonin as a therapeutic agent in the cardioprotective setting. Among them, three involved studies with myocardial infarction treated with PCI (16, 18, 19) and four used CABG setting (15, 17, 20, 21).

In 2015, Ghaeli and colleagues (16) demonstrated that oral melatonin (3 mg) administered the night following PCI and continued daily during hospitalization had neutral effects on infarction size. The authors concluded that the study was underpowered and the dosing regimen was suboptimal. In 2017, two studies provided additional data related to the effects of melatonin in patients with ST-elevation myocardial infarction (18, 19). Ekeloef and colleagues showed that 50 mg intravenous and 1 mg intracoronary melatonin at the onset of reperfusion failed to improve LVEF or reduce infarct size (18). A *post hoc* analysis of the MARIA trial demonstrated that early treatment (within 2 h) with intravenous (12 mg) and intracoronary (2 mg) melatonin resulted in less infarct area in patients with ST-elevation myocardial infarction (19).

Others studies have reported cardioprotective effects using melatonin in patients undergoing CABG (15, 17, 20, 21). Gögenur et al. with a dose of 50 mg melatonin given intraoperatively and 10 mg melatonin given orally, during elective abdominal aortic aneurysm repair, demonstrated reduced post-operative troponin elevation and cardiac morbidity (15). A dose of 10 and 20 mg of melatonin orally administered in patients who were undergoing elective CABG has previously been shown to reduce the degree of myocardial IR injury (17). Shafiei and colleagues demonstrated that 15 mg of oral melatonin reduced CABG-related cardiac injury (20). Other authors have suggested that in patients who were undergoing elective CABG, 10 mg of oral melatonin may be more effective as a cardioprotective agent when given prior to index ischemia (21).

Mao and colleagues (22) published a meta-analysis on this topic in animal studies. They demonstrated that pre-treatment with melatonin was associated with a significant lower infarct size in comparison with placebo in myocardial IR damage (weighted mean difference: −20.45, 95% CI −25.43 to −15.47, *p* < 0.001; *I*^2^ = 91.4%, *p* < 0.001). Moreover, a similar improvement was also noted in LVEF, which indicated the role of melatonin in attenuating IR injury and subsequent cardiac dysfunction (22). Our meta-analysis showed a significant increase in LVEF for melatonin-treated patients in comparison with placebo-controlled individuals. In addition, there was a significant reduction in plasma levels of cardiac troponin as a result of melatonin treatment. While the results on LVEF were consistent across all studies, a significant heterogeneity was found in the results on troponin levels. Nevertheless, all works reported lower levels of troponin in melatonin-treated patients, and thus, the inconsistency only lies in how much troponin levels can be reduced using this drug.

Due to the small number of studies, we could not develop a subgroup analysis stratified by different doses of melatonin or according to its route of administration. The observed inconsistencies may be due to these different types of treatment since there is no consensus of the optimal clinical effective plasma concentration or drug administration (7, 23). Therefore, the timing and form of drug administration and approaches for infarct size determination may also have an impact on the outcomes.

Oxidative stress is the most established basic mechanism that causes myocardial tissue damage during reperfusion insult. It is characterized by severe imbalance between exaggerated reactive oxygen species generation and corresponding antioxidant defense systems (5). Numerous studies had verified the role of melatonin in alleviating IR injury in animals and explored the potential underlying mechanisms, which mainly focused on its powerful capacity to scavenge free radicals and activate antioxidant enzymes (11, 24–26). Speculatively, the cardioprotective effects of melatonin might be largely dependent on a clinically effective distribution of the drug in the myocardial area at risk, prior to ischemia and definitely prior to reperfusion.

This meta-analysis has limitations. First, the results of our meta-analysis are based on a small number of RCTs, uncertainty regarding the blinding of participants, and a small number of patients. Second, significantly high heterogeneity in the levels of cardiac troponin may affect the interpretation of the results. Third, due to the paucity of studies available, we found a moderate degree of inconsistency; therefore, we could not perform subgroup analysis or metaregression that could shed light in the presence of inconsistency. Finally, there was an obvious weakness in the published works regarding the evaluation of infarct size. Only two RCTs (18, 19) assessed myocardial IR injury site using cardiac magnetic resonance imaging; magnetic resonance imaging is considered the gold standard for the assessment of myocardial infarction size (27).

## Conclusion

This meta-analysis revealed that melatonin administration in humans as a cardioprotective agent attenuated heart dysfunction with a favorable effect on the LVEF.

## Data Availability Statement

The original contributions presented in the study are included in the article/supplementary material, further inquiries can be directed to the corresponding author/s.

## Author Contributions

AD-R had full access to all of the data in the study and took responsibility for the integrity of the data and the accuracy of the data analysis. AD-R and PA-G: concept and design. NB-F, PA, and DH-V: acquisition, analysis, or interpretation of data. AD-R and DH-V: drafting of the manuscript. PA-G and RR: critical revision of the manuscript for important intellectual content. DH-V: statistical analysis. AD-R: obtained funding. All authors contributed to the article and approved the submitted version.

## Conflict of Interest

The authors declare that the research was conducted in the absence of any commercial or financial relationships that could be construed as a potential conflict of interest.
